# Comparison of primary and passaged tumor cell cultures and their application in personalized medicine

**DOI:** 10.37349/etat.2024.00237

**Published:** 2024-06-17

**Authors:** Vladislava V. Pipiya, Zarema E. Gilazieva, Shaza S. Issa, Albert A. Rizvanov, Valeriya V. Solovyeva

**Affiliations:** IRCCS Istituto Romagnolo per lo Studio dei Tumori (IRST) "Dino Amadori", Italy; ^1^Institute of Fundamental Medicine and Biology, Kazan Federal University, 420008 Kazan, Russia; ^2^Department of Genetics and Biotechnology, St. Petersburg State University, 199034 St. Petersburg, Russia; ^3^Division of Medical and Biological Sciences, Tatarstan Academy of Sciences, 420111 Kazan, Russia

**Keywords:** Primary cell cultures, passaged cell cultures, malignant neoplasms, immortalization, personalized medicine, test systems

## Abstract

Passaged cell lines represent currently an integral component in various studies of malignant neoplasms. These cell lines are utilized for drug screening both in monolayer cultures or as part of three-dimensional (3D) tumor models. They can also be used to model the tumor microenvironment in vitro and in vivo through xenotransplantation into immunocompromised animals. However, immortalized cell lines have some limitations of their own. The homogeneity of cell line populations and the extensive passaging in monolayer systems make these models distant from the original disease. Recently, there has been a growing interest among scientists in the use of primary cell lines, as these are passaged directly from human tumor tissues. In this case, cells retain the morphological and functional characteristics of the tissue from which they were derived, an advantage often not observed in passaged cultures. This review highlights the advantages and limitations of passaged and primary cell cultures, their similarities and differences, as well as existing test systems that are based on primary and passaged cell cultures for drug screening purposes.

## Introduction

Cell cultures have always been of significance for researchers, serving as models used to explore various methodologies, including those aimed at overcoming drug resistance in malignant neoplasms (MN). The primary goal of oncology research is to improve patient responses to anti-tumor therapy. Today, there is a global challenge in screening anticancer drugs, attributed to the inadequacy of used test systems [1]. All anti-tumor drugs, currently, undergo initial testing on two-dimensional (2D) cell models of MN, such as the NCI60 cell line panel developed by the National Cancer Institute in the USA [2]. However, 2D models have several drawbacks and significantly differ from in vivo tumors. Such difference is a reason why anti-tumor drugs that pass screening on such models do not exhibit similar effectiveness in studies on more sophisticated MN models and in vivo tumors. Most existing test systems do not reflect individual differences and factors inherent to a specific patient’s tumor [3]. Therefore, developing test systems for a specific patient based on primary cells isolated directly from biopsy material could contribute to more effective screening of anticancer drugs and serve as the foundation for a personalized approach to MN treatment.

## Primary cell cultures

The response of tumors to treatment may vary, so it is important to find an individual approach to the treatment of each patient. Therefore, it is necessary to find ways to collect and grow tumor cells from patients. This is to facilitate a more effective examination of the molecular mechanisms at work in a specific patient’s tumor [4]. It is worth noting that primary cell lines are a relatively new tool in oncology research. Cultivating primary tumor cells from patients can yield highly accurate data, aiding in the translation of in vitro results into in vivo models and, ultimately, clinical applications [5].

The process of obtaining primary cell cultures involves three stages: tumor resection, tumor dissociation, and cultivation. The method of resection varies depending on the location of the neoplasm within the organ. For example, hepatic resection uses lobar resection or segmental resection [6], while lung resection involves lobectomy or pneumonectomy [7] and so on. Preserving the tissue structure to the maximum extent is crucial, as the choice of an appropriate dissociation method depends on its integrity. Methods for tumor dissociation include enzymatic dissociation, chemical dissociation, and mechanical dissociation [5]. It should be noted that these tissue dissociation techniques are frequently employed in conjunction with one another. The advantages and disadvantages of these methods are outlined in Table 1.

**Table 1:** Advantages and disadvantages of different tissue dissociation methods

**Tissue dissociation methods**	**Advantages**	**Disadvantages**
Enzymatic [8–10]	-Extraction of a sufficient quantity of required cells. -Accessibility and widespread application. -Relatively easy removal of stromal cells and fibroblasts.	Requires the selection of specific enzymes for each type of tumor tissue.
Chemical [11, 12]	-Allows better preservation of phenotypic and genetic characteristics. -Cells divide rapidly when cultivated.	The complexity of the procedure, with the efficiency not fully established.
Mechanic [8–13]	Simplicity and accessibility, with no chemical impact on cells.	-Formation of a large number of non-viable cells. -Release of cell contents with damaged membranes into the external environment, and sample contamination.

Enzymatic dissociation allows for the separation of cells from the extracellular matrix, creating a single-cell suspension from fragmented tissue while preserving the viability and integrity of most cells [14]. Various enzymes are used for tumor dissociation, including trypsin, papain, elastase, hyaluronidase, collagenase, pronase, and deoxyribonuclease (DNase) [8, 9, 14]. These enzymes show different substrate specificities, with research indicating varying effectiveness for the dissociation of specific tissues [10].

For instance, trypsin interacts with amino acids (lysine, arginine), cleaving the carboxyl group; papain cleaves cysteine residues, disrupting disulfide bonds in peptides and proteins; elastase cleaves the carboxyl group of glycine, alanine, valine, hydrolyzing elastin; hyaluronidase initiates the hydrolysis of hyaluronic acid; collagenase breaks peptide bonds in collagen; DNase hydrolytically cleaves the phosphodiester bond in DNA; pronase, mainly composed of serine proteases, can cleave almost all amino acid bonds in any protein [15].

Choosing an enzyme for a specific tumor type depends on the properties of the enzymes mentioned above [5]. For example, collagenase and DNase are applied to tumor resection material from the intestine, liver, and kidneys, while papain is used for muscle tissue [15].

Various cations, such as Ca^2+^ and Mg^2+^, are used to maintain the integrity of the cell surface and intracellular structural matrix [10]. Chemical dissociation involves reducing the content of these cations in epithelial cells to weaken intercellular connections [11]. This is most effectively accomplished by the application of ethylenediaminetetraacetic acid (EDTA) or ethyleneglycoltetraacetic acid (EGTA) or tetraphenyl-boron complexes with potassium ions, which have been utilized for tissue dissociation in the liver, intestinal crypt cells, and solid tissue of the mammary gland [12].

Mechanical tissue dissociation involves multiple steps, including repeated cutting with scissors or sharp blades, homogenization, filtration, shaking, repeated aspiration, application of anomalous osmolar stress, or any combination of these methods. Typically, tumor samples are initially cut into small pieces (approximately 1 mm), followed by rinsing in tissue-specific media to remove loosely bound cells or debris [13]. This process enables the quick generation of a cell suspension with minimal manipulation. However, dissociating tumor tissue using mechanical methods is not proper for obtaining tumor cells for cultivation, as this method leads to a large number of dead cells secreting degradation enzymes [8].

Various studies have been conducted to understand how primary cultures resemble native tumors. For instance, Janik et al. [16] conducted a study to determine whether primary cultures of prostate and breast tissues retained the characteristics of tumor cells, by evaluating the mRNA expression of mucin-1 isoform Y (*MUC1/Y*). Although *MUC1* gene overexpression is typical for both normal epithelium and epithelial tumor cells, its isoform Y is considered tumor-specific [17, 18]. Prostate and breast tumor samples demonstrated a low average expression level of this gene, while in primary prostate cancer cells and primary breast cancer (BC) cells, it was elevated [16]. In the case of BC, the expression of human mammaglobin 1 gene mRNA (*MGB1*), a marker specific to breast tissue, was also assessed [19], showing comparable average levels in breast tissue samples and primary cultures [16]. In contrast, the gamma-synuclein gene (*SNCG*), associated with BC development, demonstrated a similar pattern to *MUC1/Y*, indicating a higher level of expression in primary cell culture compared to corresponding tumor samples [20].

Daigeler et al. [21] compared the differential expression of proapoptotic genes tumor necrosis factor (TNF)-related apoptosis-inducing ligand (*TRAIL*)-2, FAS cell surface death receptor, TNF ligand superfamily member 5 (*TNFRSF5*)/*CD40* and antiapoptotic genes proliferation and apoptosis adaptor protein 15 (*PEA15*), B-cell lymphoma 2 (*BCL2*)-related protein A1 (*BCL2A1*), nerve growth factor-β (*NGFB*), and others using microarrays in 19 primary cultures of liposarcoma after doxorubicin treatment. The outcomes demonstrated that primary cultures had a consistent level of response similar to what is observed in clinical practice across various disease stages, indicating their effectiveness in replicating the therapeutic impact of anticancer medications [21]. Lobo et al. [22] investigated the expression of renal epithelial markers and conducted transcriptional profiling of primary cell suspensions from clear cell renal cell carcinoma. Molecular analysis using Illumina Human Omni 2.5–8 single nucleotide polymorphism (SNP) arrays revealed that some primary tumor cell suspensions did not exhibit major genomic anomalies, characteristic of the tumors they originated from, while others demonstrated compliance in gene copy number changes with their original tumors. However, primary tumor cell suspensions also had genetic structure changes not observed in the original tumors: loss of the 4th chromosome and deletions in the 8th and 9th chromosomes. In comparison, commercial renal cell carcinoma cell lines 786-0 and A-498 had significantly greater gene copy number changes, compared to patient tumors [22].

At present, there are certain difficulties in isolating and cultivating primary tumor cells. For example, the challenge may not only be limited to the isolation of primary cultures (due to low-quality surgical material or cell shock from transitioning from in viv*o* to in vitro conditions) but also in maintaining cultures in an artificial environment to achieve the required number of passages and conduct complex analyses (due to early onset of in vitro senescence) [23]. It is important to cautiously utilize tumor dissociation techniques, as intensive mechanical processing may lead to the loss of morphology in epithelial cells. The chemical approach, using a mixture of enzymes, is effective, but the concentration of different enzymes for specific tissue should be optimized, as should the duration of enzymatic action on the tissue [5].

Therefore, primary cell lines make it possible to personalize cancer therapy by generating cell cultures from a patient’s tumor and conducting functional tests of chemotherapeutic drugs on live tumor cells. However, there are still problems, associated with obtaining these cells, the complexity of their isolation, and the limited ability to proliferate [24]. Solving these problems may allow the development of an improved test system for personalized medicine.

## Passaged cell lines

Passaged cell cultures originated from the work of Ehrlich and Apolant in 1905 [25]. They utilized spontaneously arising breast tumors in mice for subcutaneous transplantation of tumor tissues into healthy mice. In 1932, Loewenthal and Jahn obtained ascitic fluid containing tumor cells and named it “ascitic carcinoma of Ehrlich” [25]. Further an experimental tumor model was obtained that could be cultivated indefinitely only in vivo, and transplanted from one mouse to another [25]. But over time, cultivation technology improved, enabling the maintenance of cell cultures through passaging in culture vessels rather than transplantation between animals [26]. However, the term “passaged cell cultures” has persisted and is still used today, although the more accurate term is considered to be “transformed cell cultures”.

A passaged cell culture is obtained through the cultivation of primary cells. It consists of a single cell type that can be sequentially propagated many times. After a limited number of passages, they lose their initial properties, mutate, and die. Cell lines with a limited lifespan are diploid [6]. There are also passaged cell lines that can be propagated indefinitely; typically, they originate during transformation into tumor cells. Tumor cell lines are commonly obtained from patient tumors, although they can also be induced through the transformation by oncogenic viruses or chemical treatment. Transformed cell lines exhibit minimal resemblance to their original in vivo characteristics [27].

For a long time, immortalized cell lines have been frequently used for modeling cancer both in vitro and in vivo*.* These cell lines are usually cultivated as monolayers for in vitro analyses or xeno-transplanted into animals with weakened immune systems for in vivo studies [28–31]. Immortalization involves obtaining a stable cell line capable of unlimited division from cells with a limited lifespan in culture. In recent years, various techniques have been used to acquire immortalized cell cultures [32]. The most common methods include transfection with viral oncogenes, such as the simian virus 40 large tumor antigen (*SV40-LT*) and E6/E7 proteins of human papillomaviruses (HPV) [33], exogenous induction of human telomerase reverse transcriptase (*hTERT*) [27] and the use of irradiated mouse fibroblasts [34] or the inhibitor of Rho-associated protein kinase (ROCK) [35]. Most of these methods still pose challenges, since issues such as cellular senescence, aberrant differentiation, loss of heterogeneity, and changes in genetic profile can arise (Table 2) [36].

**Table 2:** Advantages and disadvantages of cell immortalization methods

**Cell immortalization methods**	**Advantages**	**Disadvantages**
Transfection with viral oncogenes [33, 37]	-Easy to use (high efficiency); -No alteration of cellular gene profile; -Maintained genome stability, and potential for differentiation.	It may lead to genome instability, changes in phenotypic and genetic characteristics of primary cells, and sometimes to oncogenicity.
Exogenous induction of *hTERT* [27, 38]	Effective and widely used method.	Similar to transfection with viral oncogenes.
Irradiated (inactivated) mouse fibroblasts [32]	Prolonged expansion of primary cells.	Cellular senescence after several passages.
Inhibition of ROCK [35]	Cell protection from apoptosis, accelerated recovery after cryopreservation.	Cellular senescence after several passages.

*hTERT*: human telomerase reverse transcriptase; ROCK: Rho-associated protein kinase

One of the most commonly used target fragments for inducing cell immortalization is a fragment of the *SV40-LT* gene [27]. Its integration into the target cell nucleus can lead to the inactivation of the p53 (a transcription factor regulating the cell cycle) and Rb (retinoblastoma protein) tumor suppressor proteins, thereby modulating the proliferative activity of cells and extending the lifespan of tumor cells. Nevertheless, the telomeres will progressively shorten until the cells stop dividing, and only a few cells will be capable of fully exiting the cell cycle and continuing to multiply, ultimately creating immortalized (passaged) cell lines [39].

Furthermore, genetic modification using HPV *E6/E7* genes can also immortalize a large number of different cell types [37]. The HPV E6 protein, as one of the most common transforming proteins, can induce the degradation of p53 protein [40] and increase the expression level of the viral oncogene *c-Myc* [41]. Moreover, it can also induce the expression of *hTERT* and enable cells for unlimited proliferation [38]. HPV E7 protein, in turn, can lead to the degradation of Rb protein [42].

Another method of cell immortalization is the exogenous induction of hTERT expression [27]. Telomerase is a specialized reverse transcriptase that synthesizes DNA repeats at the ends of chromosomes, called telomeres [43]. Wang et al. [44] in 1998 found that following transfection with a retrovirus-mediated exogenous *hTERT* gene, normal human mammary epithelial cells obtained a stable telomere length, a longer lifespan, less intense β-galactosidase staining (a senescence marker), and unchanged expression of plasminogen activator inhibitor (another senescence marker).

In 2010, Chapman et al. [45] developed a method for immortalizing primary cells called conditional reprogramming (CR). Recently, CR has emerged as a new next-generation tool for the long-term cultivation of primary epithelial cells obtained from almost all sources without altering the genetic profile of the primary cells. In CR, primary cells are co-cultured with inactivated mouse 3T3-J2 fibroblasts in the presence of Y-27632, ROCK inhibitor, allowing primary cells to acquire stem-like characteristics while retaining their ability for full differentiation. In just a few years of development, CR shows broad prospects for application in various fields, including disease modeling, regenerative medicine, drug evaluation and discovery, as well as precision medicine [32].

The main advantage of passaged cell cultures is the formation of a large amount of material for research, allowing for multiple repetitions of experiments [46]. Moreover, passaged cultures are easy to use and cultivate, providing long-term preservation of specific molecular and genetic characteristics [47]. Such features make passaged cell lines a reliable system for various clinical and laboratory tests, with the mentioned cell lines being the least expensive research tool.

Furthermore, evidence from many studies confirms the similarity between passaged tumor cell lines and primary cancers [48]. Drexler et al. [49] showed that mutations in the tumor suppressor protein p53 (*TP53*) gene were preserved in 53 out of 62 pairs of leukemia passaged cell lines and primary tumor cells. It has been also shown that glioblastoma cell lines retain the same homozygous deletions in the cyclin-dependent kinase inhibitor 2A (*CDKN2A*) gene, as the original tumors [50].

Wistuba et al. [51] compared various properties of 12 cell lines of non-small-cell lung carcinoma (average cultivation time of 39 months) and archived specimens of the same tumor (tissues taken from patients a while ago). A high percentage of similarity was found between passaged cell lines of lung cancer and corresponding tumor tissues in terms of morphology (100%), aneuploidy presence (100%), immunohistochemical expression of human epidermal growth factor (EGF) receptor-2 (HER2) (100%), and p53 protein (100%), loss of heterozygosity in 13 analyzed chromosomal regions (97%), mutations in *TP53* genes (67%), and transcription factor KRAS (100%). Some differences were also found: passaged cell lines had more aneuploid cell subpopulations and a higher frequency of *TP53* mutations (4 out of 10 mutations not detected in patient tumors) [51].

However, passaged cultures have several limitations related to large numbers of passages, as they progressively diverge from the original tumor they aim to model [29]. This disrupts the reproduction of molecular mechanisms, intercellular interactions, and morphological features of tumor cells [52], leading to challenges in understanding many processes that, likely, play a key role in tumor development and spread [53].

The genomic drift that results from the use of different techniques in cell culture methods and nutrient media in various laboratories can cause differences in both genotypic and phenotypic traits within the same cell line [54]. Ben-David et al. [55] performed a complete genomic characterization of 27 cell lines of estrogen receptor-negative (ER^–^) breast adenocarcinoma MCF7. Changes were observed, including the differential activation of gene expression programs, the transformation of cell morphology, and changes affecting the proliferative activity of cells. It was shown that sensitivity to drugs differs from one cell line to another, with 75% of tested drugs that strongly inhibited some of the MCF7 cell lines, being completely inactive in other MCF7 cell lines [55].

The use of passaged tumor cell lines in preclinical studies has been essential in advancing our understanding of tumor biology and in facilitating high-throughput drug screening for development [5]. However, the accumulation of genetic aberrations in passaged cell lines, occurring with an increasing number of passages, limits their clinical correlation [56, 57]. Nevertheless, the above-mentioned research results have shown that passaged cancer cell lines and their corresponding primary malignant tumors are similar in some key phenotypic and molecular characteristics [58].

## Comparative characteristics and molecular-genetic features of passaged and primary cell lines

Currently, there is a pressing need for new and more effective methods of drug screening. Passaged, or immortalized, tumor cell lines, commonly used in scientific practice, have several drawbacks. Prolonged cultivation often leads to the accumulation of new genetic aberrations in passaged cell lines, which may not accurately represent the heterogeneity of solid tumors, raising questions about the reliability of research findings. In contrast, primary tumor cell cultures have emerged as a viable alternative to passaged cell lines because they can better preserve certain properties of the tumors from which they were derived [59]. However, primary tumor cell cultures, like commercial cell lines, have their own drawbacks.

Since tumors have a complex organization, the passaged cell lines cannot fully reproduce their morphological organization and biochemical processes [60]. Certain tumor cells demonstrate increased proliferation capabilities in vitro, resulting in passaged cell lines that do not accurately reflect the full molecular heterogeneity present within the tumor [16]. As a result of long-term cultivation of passaged cell lines, cells acquire genetic mutations that can affect changes in their phenotype [54], functional state, and reactions to drugs [61].

It is known that primary tumor cells retain their original morphology for a limited number of passages, while passaged cell lines tend to change their original phenotype (for example, loss of polarity) due to the large number of passages [60]. Therefore, passaged cell lines cannot show the same results in studies as primary tumor cells.

Currently, primary tumor cells are increasingly regarded as the preferred model for in vitro research [5]. The restricted duration of cultivation inhibits the expansion of individual in vitro clones and thereby reduces the homogeneity of the population [61]. A brief overview of the advantages and limitations of the discussed tumor cell cultures is presented in Table 3.

**Table 3:** Advantages and limitations of primary and passaged cell cultures

**Cell type**	**Advantages**	**Disadvantages**
Primary tumor cell culture	-Reflection of molecular characteristics of tumor cells [59]; -Clinically significant for personalized therapy [4]; -Suitable for genetic manipulations and molecular studies specific to cancer cells of a particular patient [21].	-Need for complex nutrient environments [23]; -Probability of outgrowing of cancer cells by stromal components at early cavitation stages [46]; -Tumor heterogeneity may be lost depending on the cultivation method [5]; -Generation efficiency depends on the type and source of the biopsy [59].
Passaged tumor cell culture	-To maintain viability, standard and uncomplicated methods along with simple nutrient media can be used [47]; -Provides a large number of cells useful for comparative studies [46]; -Suitable for genetic manipulations and molecular studies specific to cancer cells [48–50]; -Lower cost [57].	-Genetic and phenotypic changes associated with the increased number of passages [27]; -Low similarity to therapeutic effects in clinical drug screening [54, 55]; -Lack of personalization [52].

Numerous studies are currently being carried out globally, focusing on unraveling the molecular-genetic mechanisms present in both primary and passaged cell lines. This is crucial for improving the current testing systems for screening anti-tumor drugs and for making cancer treatment more personalized and, therefore, more efficient.

For example, Lee et al. [62] analyzed gene expression in the MM6 cell line (monocytic commercial cell line obtained from a patient with acute monocytic leukemia) and primary malignant cells obtained from a patient with acute myeloid leukemia (AML) with t(9;11) translocation, using serial analysis of gene expression (SAGE), to determine whether the commercial MM6 cell line is suitable for studying AML. Comparative analysis showed that there was not a significant difference in gene expression between the two types of cell cultures. However, several genes of the extracellular signal-regulated kinase (ERK1/2) and mitogen-activated protein kinase (MAPK) signaling pathways, including *H-RAS*, were overexpressed in MM6 cells. Since the MM6 cell line has a gene expression profile similar to primary t(9;11) AML cells and expresses genes of the MAPK, ERK1/2, pathway including *H-RAS*, it can be used as a model for *H-RAS*-positive t(9;11) AML [62].

Dairkee et al. [63] found that, unlike cultured breast adenocarcinoma cell lines (T47D, SKBR3, BT20, etc.), primary tumor cell cultures exhibited a characteristic “limited proliferation” phenotype, including overexpression of genes associated with the transforming growth factor-β (TGF-β) signaling pathway, such as lysyl oxidase-like 1 (*LOXL1*), Runt-related transcription factor 1 (*RUNX1*), and death-associated protein kinase 1 (*DAPK1*), etc. At the core of this profile was a noticeable absence of *hTERT* expression and telomerase activity. The data obtained indicates that commonly used immortalized cell lines don’t closely resemble some aspects of tumor biology as much as primary tumor cell cultures.

Weigand et al. [64] investigated the content of vascular endothelial growth factor (VEGF)-A using ELISA in cultured breast adenocarcinoma cell lines MDA-MB-468, T47d, MCF7, HBL-100, and in a culture of primary breast tumor cells. It was found that VEGF-A levels exceeded the range of biological activity (1–50 ng/mL) in all cultures. Interestingly, the highest concentration of 37.3 ng/mL was obtained in the primary BC cell culture [64]. It can be concluded that the primary tumor cell culture is most capable of angiogenesis and more intensive growth, compared to commercial cell lines.

As a result, primary tumor cell cultures frequently provide a closer representation of the molecular and genetic mechanisms present in in vivo tumors, in contrast to commercial cell lines. Therefore, it is imperative to integrate test systems based on primary tumor cells in clinical practice for a more effective selection of cancer therapy and the study of mechanisms taking place in tumor tissue.

## Test systems based on primary and passaged cell cultures

2D in vitro tumor models are crucial for not only discovering new substances with anti-tumor activity but also for assessing their efficacy [3]. By the end of the 1980s, an in vitro panel for drug screening was developed, and named NCI60, consisting of 60 different human cell lines derived from tumors (leukemia, melanoma, tumors of the central nervous system, lung cancer, colon cancer, ovarian, breast, kidney, and prostate cancers) [1].

There are test systems similar to NCI60, representing a panel of specific (homogeneous) tumor lines, such as the BC cell line panel and the colorectal cancer cell line panel [65]. However, 2D models have several significant drawbacks. In regular 2D cultures, cells grow rapidly, demonstrate unnatural morphology, have lower viability and poor differentiation, and do not show the same reactions as tumors in vivo*.* However, the ease of use and cost-effectiveness of cell lines make them a convenient model for studying anti-tumor drugs and the properties of tumor cells in general [3].

Another test system is the Boyden chamber, initially designed to study leukocyte chemotaxis. Boyden chamber has become one of the most frequently used methods for assessing cell mobility and invasion [66]. The traditional Boyden chamber is composed of two compartments divided by a membrane, acting as a physical obstacle that cells can only traverse through active migration [67]. The Boyden chamber can be modified to examine the invasive characteristics of tumor cells by applying diverse extracellular matrix proteins onto the membrane [68].

Richbart et al. [69] developed a new method for studying the invasive activity of tumor cells (spherical invasion assay), based on the application of the Boyden chamber. The anti-invasive activity of the Src kinase inhibitor (non-related to tyrosine kinase receptor) PP2 was measured in human non-small cell lung cancer (NSCLC) A549 cells. However, Richbart et al. [69] observed that the findings of the spherical invasion assay need to be validated through the Boyden chamber invasion analysis. By integrating the outcomes of both analyses, it could be feasible to ascertain if the candidate drug demonstrates anti-invasive properties. Spherical invasion assay can be adapted for several experimental constructs, such as drug screening (to combat invasion and metastasis), measuring the pro-invasive activity of growth factors, and identifying signaling pathways underlying the pro-invasive and anti-invasive activity of biological modifiers [69].

More modern methods can be used to investigate invasion and migration, which include the use of the xCELLigence device and the Transwell method. Impedance changes in a network of gold microelectrodes, either on the bottom of a well or on a microporous membrane, can be measured using xCELLigence technology. These changes occur as cells proliferate, migrate, or invade the electrode surface over time. This method of quantitation is correlated with cellular morphology, spreading, ruffling, adhesion quality, and cell number [70, 71].

Another method, the Transwell invasion assay utilizes multi-well plates made of disposable plastic with a microporous membrane [72]. Cells can be observed using fluorescent staining or fixed and stained with crystal violet. The number of migrating cells is determined by the percentage of cells that have traversed the membrane toward the chemoattractant. This method offers high sensitivity, as even a low concentration of chemoattractant can stimulate cell migration through the membrane. However, the duration of the study is limited due to the quick equalization of concentrations between compartments [73].

In addition, various modifications of this method can be carried out. One such method is the Transwell-Hypoxia model, which offers an enhanced alternative approach for studying tumors in vitro, eliminating the need for animal experimentation. It was found that the Transwell-Hypoxia method better mimics the tumor microenvironment using cryopreserved patient-derived melanoma explants [74].

## Three-dimensional in vitro cell culture systems

Currently, many 3D in vitro cell culture systems have been developed to approximate the growth conditions of tumors in vivo. Techniques for cultivating tumor cells in 3D are designed to maintain the biological features of tumors in vivo, which encompass multicellular tumor spheroids (MCTs), organotypic multicellular spheroids, organoids derived from tumors and assembloids [75].

### Spheroids

MCTs are usually cultivated from commercial tumor cell lines in standard cultures, similar to standard monolayer cultures. The main difference from 2D cultures is that MCTs cells are cultured as spheres formed from a cell suspension under special conditions (e.g., under the influence of gravity in the “hanging drop” method), which promotes cell adhesion [76]. Despite the fact that MCTs have extremely low histological similarity to tumors in vivo, cells in MCTs mimic the metabolic and proliferative gradients of tumors in vivo and, unlike monolayer cell cultures, demonstrate clinically significant chemoresistance [77].

Organotypic tumor spheroids are 3D model created from the tumor cells of a patient’s biopsy in vitro, unlike MCTs, which use passaged cell lines. These spheroids preserve the heterogeneity and structural characteristics of the tissue, which makes them a more accurate imitation of a tumor in vivo than traditional 2D and 3D models of cell lines. These structures contain tumorigenic cells, as has been proven by studies on animal models with xenograft implantation, and preserve the genetic composition of the original tumor material [78].

Melissaridou et al. [79] investigated five cell lines of head and neck squamous cell carcinoma (HNSCC) to assess the effects of 3D cultivation, compared to 2D monolayers, regarding cell proliferation, response to anti-tumor therapy, and the expression of epithelial-mesenchymal transition (EMT) and cancer stem cell genes. It was found that all spheroids exhibited increased regulation of cadherin 1 (*CDH1*), *NANOG*, and *SOX2* (transcription factors of stem cells) compared to 2D cultures, but changes in the expression of EGF receptor (EGFR) and EMT markers varied between cell lines. Moreover, most HNSCC cells grown in 3D culture showed reduced sensitivity to cisplatin and cetuximab (anti-EGFR treatment) [79].

### Organoid cultures

Over the last decade, different methods have been developed for growing tissues in vitro in 3D culture as organotypic structures. Organoids, which can originate from both differentiated and embryonic stem cells, possess the capacity to autonomously form into 3D structures that mimic the architecture of the tissue of origin (in the case of organoids derived from adult stem cells) or the tissue to which differentiation was directed (for organoids derived from embryonic stem cells) [80].

Broutier et al. [81] found that primary organoid cultures of liver cancer, obtained from patients, could be cultured for a long time in vitro, maintaining the histological architecture of the tumor subtype from which they originated. The first hierarchical cluster analysis, comparing gene expression profiles of tissue samples with publicly available data from the Cancer Genome Atlas Primary Liver Cancer (TCGA-PLC), confirmed that the samples used in this study were representative of the general population of primary liver cancer. Therefore, it was established that tumors replicate the gene expression profiles of the specific tissue of origin. These results show that the organoid cultivation system derived from primary liver cancer accurately replicates and corresponds to transcriptomic changes present in the tumor subtype of individual patients. As all diverse tumor subtypes were kept under identical cultivation conditions, these findings imply that their tumor signature is ingrained in the cancer population and remains largely unaffected by cultivation conditions [81].

Furthermore, numerous BC research studies are currently using organoid cultures. It is known that a representative organoid model, besides histological similarity, should maintain the expression of the most important and common BC biomarkers: ER, progesterone receptor (PR), and HER2 [82]. The status of hormonal receptors ER and PR has prognostic value for the outcome of hormonal/endocrine therapy in BC, while HER2 status can predict the outcome of systemic chemotherapy and is itself a target for cancer therapy [83].

Sachs et al. [82] found that the hormonal receptor and HER2 status were maintained in most pairs of organoid systems and the original tumor, as determined by immunohistochemical analyses. ER- and/or PR-positive tumors resulted in ER- and/or PR-positive BC organoids in approximately 75% of cases. ER- and/or PR-negative tumors generated > 90% ER- and/or PR-negative BC organoids. The HER2 status was preserved in 80% of HER2-positive and > 90% of HER2-negative pairs of organoids and the original tumor. Thus, it was established that most BC organoids correspond to the original tumor in terms of histopathology and the presence of hormone receptors and HER2. However, histological analysis did not plainly assign a tumor status to all BC organoids; some organoids were classified either as well-differentiated tumors or as organoids derived from normal epithelial cells of the mammary gland [82].

All the mentioned above indicate that the organoid test system is a promising model for researching tumors and screening drugs, as it closely replicates the histological features of the original tumor, and the transcription of key genes and various clinically important tumor receptors.

### Assembloids

Currently, a model such as assembloids can be used to study tumors. Assembloids are 3D systems of cell cultures that are formed as a result of the joint cultivation of one type of organoid with another organoid or with specialized cell cultures. Thus, assembloids are a self-organizing 3D in vitro culture system that contains a set of specialized cell types and reproduces some function of the organ or part of it [84]. There are several types of assembloids: multi-region, multi-lineage, inter-individual, and inter-species assembloids.

Multi-region assembloids may contain several types of organoids, which may consist of different cell cultures (for example: human induced pluripotent stem cells, human embryonic stem cells, or isolated adult stem cells). At the same time, multi-lineage assembloids are obtained from specialized stem cells, cultured together with organoids. Inter-individual assembloids are cultivated from organoids originating from different individuals of the same species. Such assembloids are used to study cellular effects. Other assembloids obtained by combining organoids of different species (for example, humans and chimpanzees) are called inter-species assembloids [85].

Thus, Lv et al. [86] developed a model of peritoneal metastasis colorectal adenocarcinoma (CRC) in vitro based on micro vascularized tumor assembloids for drug screening. It has been found that such an assembloids model can support a pattern of gene expression, as well as a similar xenograft model. In addition, perfusion of antitumor drugs in vitro during hyperthermic intraperitoneal chemotherapy can mimic the perfusion of the drug during chemotherapy in vivo [86].

Kim et al. [87] developed assembloids by culturing four organoids of a bladder tumor: two luminal and two basal. These assembloids also included tumor-associated fibroblasts obtained from the patient and endothelial cells. Morphological changes in tumor growth were observed in assembloids, which showed phenotypic similarity to xenografts and parent tumor tissues. It was also found that genetic changes in parental tumors persist in both organoid models and tumor assembloids [87].

Sharpe et al. [88] created an assembloid model consisting of esophageal adenocarcinoma organoids and tumor-associated fibroblasts. Cell phenotypes were evaluated using immunofluorescent and histological methods. It was found that the assembloid models accurately reflect the differentiation of esophageal adenocarcinoma cells and tumor-associated fibroblasts [88].

Thus, such models more accurately recreate tumors with the potential to yield results that could lead to the discovery of new tumor therapy methods and the development of innovative approaches in medicine. Due to the complex structure and functional diversity of tumor assembloids, their use can aid in forming a deeper understanding of the causes of tumors and ways to combat them. In the long term, such test systems could become an important tool in developing personalized approaches to tumor treatment.

## Patient-derived xenografts

There are test systems that facilitate the study of processes occurring in the tumor and its microenvironment inside a living organism. Patient-derived xenografts (PDX), where tumor fragments surgically extracted from patients with MN are directly transplanted into immunodeficient mice, have become a valuable model for translational research aimed at developing personalized medicine [89, 90]. The susceptibility of PDX to anti-cancer drugs closely correlates with clinical data from the patients they were derived from, indicating the potential use of PDX models in predicting the effectiveness of both conventional and new anti-tumor therapeutic agents [91, 92].

However, the heterogeneity of the tumor present in PDX models and in the original tumor samples introduces an obstacle to the application of PDX models [89]. Moreover, human stromal cells initially present in tumors obtained from patients are gradually replaced by host organism stromal cells as the xenograft grows. This replacement may cause changes in the paracrine regulation of the tumor and lead to the disruption of the distribution of drugs [80]. This mouse stromal replacement may impede the study of the interaction between the human tumor and stroma, as some mouse stromal cytokines may influence human carcinoma cells in PDX models [93].

Misale et al. [91] treated patients with progressive CRC with EGFR antibodies, after the initial response to EGFR inhibitors had led to disease recurrence due to emerging resistance. Therefore, they investigated how the risk of resistance to EGFR-targeted therapy in CRC tumor cells could be reduced using a PDX model. Genetic screening in vitro and functional studies showed that dual blockade of EGFR and MEK prevented acquired resistance. This research also conducted in vivo experiments using PDX derived from a patient with CRC carrying four wild-type genes [without mutations, *BRAF*, *KRAS*, *NRAS* (genes of signaling proteins, members of the RAS family), and *PIK3CA* (encodes the p110α catalytic subunit of phosphatidylinositol 3-kinase (PI3K))], which replicated the gene expression profile of patients sensitive to anti-EGFR antibodies. While treatment with the MEK inhibitor pimasertib only slightly reduced tumor growth, treatment with the EGFR inhibitor cetuximab effectively reduced cancer proliferation by more than 70%.

It has been established that PDX models retain essential properties of patient-derived tumors, including metastatic features, making them physiologically significant for studying metastatic properties of tumor cells [94]. Using the PDX model, Lawson et al. [95] demonstrated that progression to high metastatic load is associated with increased proliferation and expression of *Myc* (a transcription factor of stem cells), which can be suppressed by treatment with CDKN. In this study, PDX with the highest metastasis had the highest percentage of tumor stem cells, while PDX with lower metastasis had the lowest. This suggests that primary tumors contain a rare subpopulation of stem cells, and the relative abundance of these cells may correlate with the metastatic potential.

## Other methods of studying tumor

Another modern method of studying the oncogenic process is the creation of test systems based on microfluidic devices. Microfluidic systems are promising cellular models needed to reconstruct the migration, microenvironment, and microcirculation of cells in tumor tissue. Microfluidic systems are small devices that can reproduce specific fluid flow, constant temperature, fresh environment, flow pressure, and chemical gradients characteristic of in vivo systems [3]. These platforms provide effective drug screening, require a small sample volume, create a cellular microenvironment comparable to in vivo conditions, and solve ethical problems. Multiple cell cultures in microfluidic chips can better simulate the 3D environment of tumors with reduced reagent consumption [96].

Anguiano et al. [97] applied a microfluidic system using collagen-Matrigel hydrogel matrices, allowing reproduction of the microenvironment and experimental conditions to study migration and invasion of lung adenocarcinoma H1299 cells. At the same time, Matrigel (a mixture of proteins secreted by Engelbreth-Holm-Swarm mouse sarcoma cells), at low concentrations, promoted the migration of H1299 cells, but at high concentrations, Matrigel slowed cell migration, possibly due to their excessive attachment. It was also shown that the use of antibody-based integrin blockers significantly modulated the migration mechanisms of H1299 cells [97].

The “tumor-on-a-chip” systems, which are microfluidic devices aimed at recreating relevant features of tumor physiology, have recently become powerful tools in cancer research. Patra et al. [98] cultivated human hepatocellular carcinoma cells (HepG2) as spheroids of two different sizes in microfluidic devices using polydimethylsiloxane. These tumor chip systems had several rectangular reservoirs or chambers at the bottom of the microfluidic channel where the spheroids would be placed. The authors assessed the impact of cisplatin, a widely used anticancer agent, on spheroids of different sizes. They also conducted similar experiments in 2D systems and confirmed that the cell culture format and spheroid size were key determinants in the observed reactions to the drug. Cisplatin exhibited its highest apoptotic activity in small spheroids. Surprisingly, even with the addition of a relatively high concentration of the drug, only about 30% of tumor cells within the spheroids died. 2D cultures were significantly more susceptible to cisplatin [98].

Lim and Park [99] developed a tumor-on-a-chip device in combination with a concentration gradient generator, to assess the effectiveness of anticancer drugs on spheroids. Spheroids of CRC HCT116, approximately 120 μm in size, were cultured in this device and perfused for 3 days in the presence of an irinotecan gradient, an inhibitor of topoisomerase I. With an increase in drug concentration (0 μM, 1.25 μM, 2.5 μM, 3.75 μM, and 5 μM), there was progressive disruption of the spheroid structure, a decrease in their number, a change in the shape of the spheroids (they became less round), and a reduction in the viability of tumor cells constituting the spheroids. This “tumor-on-a-chip” system demonstrated an important microfluidic feature for cancer research, namely the ability to generate gradients [99].

Shirure et al. [100] created a “tumor on a chip” model that simulates the transport of drugs through the vascular network. A breast tumor cell culture and tumor organoids cultured from primary tumor cells of patients were used as a tumor model. The use of this model makes it possible to study the proliferative ability of tumor cells, their migration through blood vessels, as well as the process of formation and growth of blood vessels [96, 100].

Therefore, when applied to personalized medical approaches, this simple device could be a convenient tool for the parallel analysis of the effectiveness and safety of cancer drugs and for determining the correct drug dose for specific patients. However, it is worth noting that in vitro screening systems for anticancer drugs cannot accurately reflect the drug’s effectiveness in vivo without mimicking the tumor microenvironment, which includes tumor cells interacting with blood vessels and fibroblasts. Accordingly, a new, more advanced method, 3D bioprinting, has been developed.

Han et al. [101] used bioprinting to construct a vascularized tumor microenvironment. Subsequently, MCTs of U87 MG glioblastoma cells were seeded onto the layer of blood vessels and incubated until endothelial cells in the blood vessel layer migrated into the MCTs, inducing angiogenesis. They found that combined treatment, involving the anticancer drug temozolomide and the angiogenesis inhibitor sunitinib, was more effective than temozolomide alone for MCTs surrounded by blood vessels, indicating the relevance of the tumor microenvironment for testing drug effectiveness in vitro [101].

Therefore, for the most accurate screening of anticancer drugs, it is of great importance to use systems as close as possible to in vivo tumors. However, there are still many limitations, such as cost, complexity, and the considerable amount of time required to create such systems, which prevent some of them from being widely implemented in preclinical and clinical trials. Despite the drawbacks of some test systems, researchers still strive to apply them in clinical studies, and test systems based on primary tumor cells are increasingly being used (Table 4).

**Table 4:** Clinical studies using test systems based on primary tumor cells (the information was obtained using the website ClinicalTrials.gov)

**Clinical trial identifier**	**Estimated enrollment**	**Cancer type**	**Cell culture type**	**Purpose**
NCT04298489	40	Gastrointestinal	Primary cell	Explore the clinical feasibility of using the primary cell culture system to guide gastrointestinal cancer chemotherapy (5-fluorouracil, oxaliplatin, irinotecan).
NCT04797676	20	Pancreatic ductal adenocarcinoma	Primary cell	Investigate the difference in the success rate of culturing primary cells obtained by endoscopic ultrasound-guided fine-needle biopsy wet suction technique and surgery.
NCT04342286	20	Kidney cancer	Primary cell (organoid culture)	To establish a sustainable human kidney tumor 3D Matrigel culture system with a stable phenotype.
NCT05813509	30	Ovarian cancer	Primary cell (organoid culture)	Use organoids cultured from patients’ own ovarian cancer tissues as models, and screen potential clinical therapeutic drugs (paclitaxel, carboplatin, lobaplatin, doxorubicin, etc.).
NCT02646228	500	Metastatic cancer	Primary cell	Establishment of patient-derived cancer cell models to interrogate novel molecular targets in metastatic cancer.

## Conclusion

Although not perfect model systems, cell lines possess numerous advantages that supplement experimental data obtained using tumor tissues and other models of oncological diseases. Without cell lines, our knowledge of tumors, their origins, and treatments would be significantly less extensive. Cell lines remain and will continue to be invaluable tools for numerous scientific discoveries. However, it is crucial to know how closely the model used in the study resembles the original tumor, and whether it has significant mutations that can impact the experiment’s results. Understanding these factors allows for the assessment of in vitro models and the ability to interpret obtained data.

For decades, passaged tumor cell lines have constituted an accessible, convenient set of models for studying cancer biology and the potential effectiveness of anticancer drugs. However, numerous studies show that passaged cell lines poorly reflect the diversity, heterogeneity, and drug resistance of tumors, encountered in patients. Therefore, obtaining and short-term cultivation of primary cells from solid tumors, have gained significant importance in personalized therapy for oncological diseases. In turn, primary cancer cells represent a favorable choice for in vitro oncology research since they, more accurately, reflect the tumor’s state, in comparison with most commonly used passaged cell lines. Unfortunately, due to the limited availability of material and difficulties in validating protocols, primary cell cultures are rarely introduced into laboratory practice. Perhaps, in the future, the development of new methods for obtaining and cultivating primary cell cultures will allow the full utilization of these models to create anticancer drug screening systems.

## Abbreviations

2D: two-dimensional
AML: acute myeloid leukemia
BC: breast cancer
CR: conditional reprogramming
CRC: colorectal adenocarcinoma
DNase: deoxyribonuclease
EGFR: epidermal growth factor receptor
ER^–^: estrogen receptor-negative
HER2: human epidermal growth factor receptor-2
HPV: human papillomaviruses
hTERT: human telomerase reverse transcriptase
MCTs: multicellular tumor spheroids
MN: malignant neoplasms
PDX: patient-derived xenografts
PR: progesterone receptor
ROCK: Rho-associated protein kinase
TNF: tumor necrosis factor
TP53: tumor suppressor protein p53

## Declarations

### Copyright

© The Author(s) 2024.
